# IMPLEMENTATION FRAMEWORKS IN ACTION: A QUALITATIVE STUDY OF INTERGENERATIONAL TECHNOLOGY IN HIGHER EDUCATION

**DOI:** 10.1093/geroni/igad104.1445

**Published:** 2023-12-21

**Authors:** Jill Juris, Rachel Scrivano, Skye Leedahl, Shannon Jarrott

**Affiliations:** Appalachian State University, Boone, North Carolina, United States; Boston College, Boston, Massachusetts, United States; University of Rhode Island, Kingston, Rhode Island, United States; The Ohio State University, Columbus, Ohio, United States

## Abstract

Cyber-Seniors is an intergenerational technology program that bridges the digital divide by training younger people to assist older adults with using technology. While higher education is an ideal setting for utilizing this program for service learning or student internship opportunities, there are often challenges to implementing this program in a higher education setting. In this study, implementation factors must be studied to improve sustainability and increase the potential to scale-up the use of Cyber-Seniors programming within higher education. This study is guided by the dynamic sustainability framework (Chambers et al., 2013) with the goal to investigate prominent contextual factors of implementation that permit and prohibit program sustainability. The study will also identify needed adaptations to Cyber-Seniors’ implementation, which will be documented using Stirman and colleagues’ (2019) Framework for Reporting Adaptations and Modifications-Enhanced (FRAME). In this presentation, we will illustrate the process of infusing implementation science frameworks into a multistage qualitative study designed to allow for in-depth understanding of experiences implementing Cyber-Seniors programming in higher education and designing an adapted checklist for program implementation. We will describe how the implementation science frameworks have been used to design the research instruments, protocols, and the analysis plan. The presentation will conclude with next steps for the research project. A key takeaway from this presentation will be for attendees to gain an understanding of how to infuse implementation science frameworks when designing, implementing, and evaluating research programs and interventions.

